# Bibliographical Notices

**Published:** 1844-09-07

**Authors:** 


### BIBLIOGRAPHICAL NOTICES.

*Principles of Pathology and Practice of Medicine.* By John Mackintosh, M. D., Lecturer on the Practice of Physic in Edinburgh, &c. &c. &c. *Fourth American, from the last London edition. With Notes and Additions, by* Samuel George Morton, M. D., formerly Physician to the Philadelphia Hospital; Author of Illustrations of Pulmonary Consumption, &c. &c. Octavo, pp. 892. Philadelphia, Lindsay and Blakiston. 1844.

The work of Dr. Mackintosh has been so long and extensively before the profession, that it is only necessary to announce the publication of a new edition of it, unde the careful revision of a highly competent American ed tor.

"The high estimation in which Dr. Mackintosh's Practice of Medicine is held, both in this country and in Europe," the Editor justly observes, "is sufficiently attested by the fact that it is now published for the eighth time, four editions having appeared in England and four in the United States."

The contributions by the Editor are numerous and practical, and add greatly to the value of the book; which, for the reader's comfort, as well as for the credit of the publishers, we are pleased to remark is brought out in good style—the binding, paper, and typography, are just such as we should desire for a book of every day reference.

---

*A System of Human Anatomy, General and Special.* By Erasmus Wilson, M. D., Lecturer on Anatomy, London. *Second American Edition.* Edited by Paul B. Goddard, A. M., M. D., Lecturer on Anatomy, and Demonstrator in the University of Pennsylvania. &c. &c. With over two hundred illustrations by Gilbert. From the second London Edition. Octavo, pp. 607. Philadelphia, Lea and Blanchard. 1844.

This is an excellent work, particularly well adapted for the use of students of medicine as a text-book, and as a book of reference for practitioners. That it is well appreciated by the profession is shown by the fact, that in less than four years since its first publication, it has passed through two editions in England, and has now attained to a second in this country.

The Editor is advantageously known as connected with the republication of various medical works, and as a practised and skilful anatomist. "In preparing the present edition," he remarks, "no pains has been spared to render it as complete a Manual of Anatomy for the medical student as possible. A chapter on Histology has therefore been prefixed, and a considerable number of new cuts added. Among the latter are included some very fine ones of the nerves, which were almost wholly omitted from the original work. Great care has also been taken to have this edition correct, and the cuts carefully and beautifully worked, and it is confidently believed that it will give satisfaction, offering a further inducement to its general use as a Text-Book in the various colleges."

The mechanical execution of the work is in the excellent style that characterizes all the recent medical publications from the well known house of Lea & Blanchard.

---

*Anatomical Atlas illustrative of the Structure of the Human Body.* By Henry H. Smith, M.D., &c. &c. Uuder the supervision of William E. Horner, M.D., &c. &c. Part 3. Royal 8vo. Philadelphia. 1844.

The third part of this Atlas, which contains illustrations of the Organs of Digestion and Generation, is deserving of all the commendations which we passed upon its predecessors. Most of the engravings, which are *one hundred and ninety-one* in number, are well executed; some few not so sharply defined as we should like to see them. The work, when completed—and the remaining parts will appear shortly—will be a valuable accompaniment to the anatomical student. It need hardly be said that typographical accuracy is all important where the instruction of the tyro—too apt to be negligent in his orthography—is the great object. A few accidental errors we notice: for example, *frœnum, filliformis, coledichus, Lieberkuhn, Bruner, Pons Hepatilis, symphysis pubes* and *vassa,* all clearly errors of the press. We might also question the necessity or the propriety of the expression 'my*loid* attachment' of a muscle; of *Hepatico-gastric* for *Hepato-gastric,* and of *Couper's glands* for *Cowper's*—the distinguished anatomist after whom they were named being William Cowper.

We think, too, in giving many of the microscopic appearances of tissues, the name of the observer should have been mentioned. Some of them can scarcely be regarded as established additions to the science. This is, however, a point on which there may be an honest difference of sentiment.

---

*Sicheres Heilverfahren bei dem schnell gefährlichen Lufteintritt in die Venen und dessen gerichtsärztliche Wichtigkeit.* Von Dr. Ch. Jos. Edl. v. Wattmann, u. s. w. Regierungsrathe, Leibchirurg, u. s. w. mit einer chilographischen Tafel. 8vo. S. 188. Wien: 1843.

*A Certain method for curing the highly dangerous entrance of air into the veins; and the medico-legal importance of the same.* By Dr. Ch. Jos. Edl. von. Wattmann, &c. &c.

Some years ago the entrance of air into the veins excited a degree of interest, which is at present subdued, but not destroyed. Professor von Wattmann had several opportunities for watching closely and accurately the spontaneous entry of air into the internal jugular, and was fortunate enough to discover, in the very first case, effectual means for saving the patient. He observed, that a whizzing sound marked the entrance of air into the wounded vein; and he found that the danger of instantaneous syncope and speedy death might be prevented by promptly closing the opening by a slight pressure of the finger; and that it could be best united and cicatrized by a ligature applied to the side of the vessel, so that the circulation through it might not be interrupted. The whole procedure is described in the work before us, which, moreover, contains a good account of the cases recorded by various observers.

